# Predictors of Post-Stroke Depression: A Retrospective Cohort Study

**DOI:** 10.3390/brainsci12080993

**Published:** 2022-07-27

**Authors:** Durgesh Chaudhary, Isabel Friedenberg, Vishakha Sharma, Pragyan Sharma, Vida Abedi, Ramin Zand, Jiang Li

**Affiliations:** 1 Department of Neurology, Neuroscience Institute, Geisinger Health System, Danville, PA 17822, USA; durgesh.chaudhary@outlook.com; 2 Department of Neurology, College of Medicine, The Pennsylvania State University, Hershey, PA 17033, USA; ramin.zand@gmail.com; 3 Lewis Katz School of Medicine, Temple University, Philadelphia, PA 19140, USA; bellafriedenberg@gmail.com; 4 College of Osteopathic Medicine, Kansas City University, Kansas City, MI 64106, USA; vishakha.sharma@kansascity.edu; 5 Department of Psychiatry, University of New Mexico School of Medicine, Albuquerque, NM 87121, USA; pragyan.sharma.md@gmail.com; 6 Department of Public Health Sciences, College of Medicine, The Pennsylvania State University, Hershey, PA 17033, USA; vidaabedi@gmail.com; 7 Department of Molecular and Functional Genomics, Geisinger Health System, Danville, PA 17822, USA

**Keywords:** stroke, ischemic stroke, post-stroke depression, predictors

## Abstract

Despite reports of a high incidence and various predictors of post-stroke depression (PSD), the underdiagnosis and undertreatment rates of PSD are still high. This study aimed to examine the incidence of depression in stroke patients and identify factors associated with PSD. This was a retrospective cohort study on ischemic stroke patients from the Geisinger Neuroscience Ischemic Stroke (GNSIS) registry. The following statistical analyses were performed to predict PSD in the studied population: a Kaplan–Meier estimator and a Cox proportional hazards model. A total of 5882 patients were included in the study. The median age at the time of an ischemic stroke was 72 years and 56% were males. A total of 294 patients were diagnosed with PSD within one year of a stroke. The cumulative incidence of depression was found to be 6.4% (95% CI 5.7–7.1%) at one year for the entire cohort. Women were found to have a higher risk of PSD than men (HR for women = 1.47, 95% CI 1.18–1.85, *p* = 0.001). A history of prior stroke (HR = 1.58, 95% CI 1.18–2.11, *p* = 0.002) and myocardial infarction (HR = 1.47, 95% CI 1.05–2.06, *p* = 0.025) were associated with PSD. Medicaid patients had a higher risk for PSD (HR = 2.16, 95% CI 1.5–3.12, *p* < 0.001) than those with commercial insurance or health maintenance organization plans. Our findings showed that women, patients with a history of prior stroke or myocardial infarction, and with Medicaid insurance were more likely to develop PSD. Through an observational study on the EHR data, we confirmed that chronic stress, including financial and health-related stress, irrespective of age, significantly increased the risk for PSD.

## 1. Introduction

Stroke is the second leading cause of mortality worldwide and the leading cause of disability [1]. As of 2019, in the United States, the American Heart Association (AHA) estimated that about 7 million people had a stroke [2,3], with an annual rate of about 800,000 [3]. A stroke may increase the risk of a variety of psychiatric disorders, such as depression, anxiety disorder, psychosis, and obsessive-compulsive disorder [4,5,6,7,8]. Depression after a stroke is a severe complication, with a reported incidence ranging from 20% to 43% at different follow-up periods [9,10,11,12,13]. Post-stroke depression (PSD) is continuously underdiagnosed and undertreated, despite its high prevalence [14]. Furthermore, the etiology of PSD is still poorly understood [15].

Most pioneer studies on PSD followed up patients for six to twelve months [10,16,17,18,19] and a few studies followed up patients for more than two years after an initial stroke [9,20,21,22]. From the results of these short-term follow-up studies, the presence of PSD appears to be time-dependent, with a higher incidence rate immediately following the event [9,16,19]. However, an increase in the incidence of PSD was seen between two months and one year in one study rather than a decrease over time [10]. These discrepancies warrant further research using large and comprehensive datasets.

Furthermore, the demographic and clinical risk factors contributing to PSD have not reached a consensus. Several studies found PSD to be more prevalent in women [16,20,23]. However, one study contradicted this finding [24]. Some studies emphasized more specific risk factors associated with PSD; for instance, patients experiencing stroke recurrence have an increased rate of PSD [16]. Patients with PSD may also experience a higher rate of mortality [21]. Therefore, further examination of gender, age, and comorbidities may lend more insight into preventative care and personalized management for patients at-risk of PSD.

The aims of this retrospective cohort study were to (1) determine the incidence of depression in ischemic stroke patients up to one year post-stroke and (2) identify the associated risk factors.

## 2. Materials and Methods

### 2.1. Study Design and Population

This retrospective cohort study included patients from an ischemic stroke registry named Geisinger Neuroscience Ischemic Stroke (GNSIS) database, which was built using Geisinger’s Electronic Health Record (EHR) data, the Geisinger Quality database, Geisinger Health Plan claims data, and the Social Security Death Index. Geisinger is a fully integrated health system that serves central, south-central, and northeastern Pennsylvania (USA). The GNSIS data includes demographic, clinical, laboratory, and imaging data from 8929 ischemic stroke patients between September 2003 and May 2019. Patients were included in the GNSIS database if they had a primary discharge diagnosis of ischemic stroke using the International Classification of Diseases, Ninth Revision or Tenth Revision, Clinical Modification (ICD-9-CM/ICD-10-CM) codes; a brain magnetic resonance imaging (MRI) to confirm the diagnosis; and an overnight stay in the hospital. Further details of the GNSIS registry were outlined in earlier publications [25,26,27].

### 2.2. Cohort Inclusion/Exclusion Criteria

**Figure 1:**
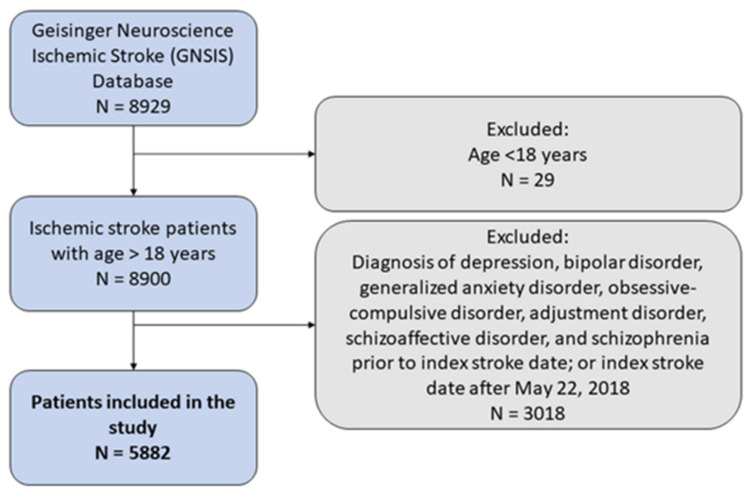
Flowchart of patients included in the study.

Patients in GNSIS with an index ischemic stroke date until 22 May 2018 and those more than 18 years of age at the time of the stroke were included in the study. Patients that were less than 18 years of age, had an index stroke date after 22 May 2018, or had a history of depression, bipolar disorder, generalized anxiety disorder, obsessive-compulsive disorder, adjustment disorder, schizoaffective disorder, and schizophrenia prior to the index stroke were excluded (Figure 1).

### 2.3. Assessment Measures or Recorded Variables

The variables of the patients that were extracted from GNSIS included demographic variables, such as gender and age at the diagnosis of the stroke. Comorbidities included in this study were atrial fibrillation, hypertension, myocardial infarction, diabetes, dyslipidemia, congestive heart failure, hypercoagulable states, chronic liver disease, chronic lung disease (asthma, chronic obstructive pulmonary disease, occupational lung disease), rheumatic diseases, chronic kidney disease, neoplasm, perivascular disease, and prior stroke history. Social and socioeconomic variables included in the study were a history of smoking, drug abuse or dependence, and the type of health insurance.

### 2.4. Outcome Definition and Follow-Up

The outcome of interest in this study was a post-stroke diagnosis of depression. A psychiatrist makes a primary diagnosis of depression based on the signs and symptoms of depression and the duration of symptoms. Then, ICD-9-CM/ICD-10-CM codes are assigned after a primary diagnosis of depression is made. The “Rule of Two” was used to identify patients with PSD, i.e., the presence of an ICD-9-CM/ICD-10-CM code for depression needed to be present in at least two separate encounters, and the date of the first qualifying encounter was used as the outcome date. The ICD-9-CM codes used in the diagnosis of PSD were 296.20, 296.21, 296.22, 296.23, 296.24, 296.25, 296.26, 296.30, 296.31, 296.32, 296.33, 296.34, 296.35, 296.36, 300.4, or 311. The ICD-10-CM codes used in the diagnosis of PSD were F32.0, F32.1, F32.2, F32.3, F32.4, F32.5, F32.8, F32.9, F33.0, F33.1, F33.2, F33.3, F33.41, F33.42, F33.9, or F34.1. The follow-up period was from the date of index stroke encounter until the last recorded encounter date of the patient in the EHR for a maximum of one year.

### 2.5. Statistical Analysis

For the study population, categorical variables were summarized as count and percentage, and continuous variables as mean ± standard deviation (SD) or median with interquartile range (IQR). For comparison between the two groups, the Pearson’s Chi-squared test was employed for categorical variables, and the Mann–Whitney U test was used for continuous variables. A Kaplan–Meier estimator with a log-rank test was used to plot the cumulative incidence of the outcome of interest after the index stroke date. A stratified Cox proportional hazards model with backward elimination was employed to examine the factors associated with the outcome of interest after the index stroke date. For all analyses, *p* < 0.05 was considered significant. All statistical analyses were performed in R version 4.0.3.

## 3. Results

### 3.1. Sample Characteristics

A total of 5882 ischemic stroke patients from GNSIS were included in this study. The median age at the stroke diagnosis was 72 (IQR 61.7–81.2) years, and 3296 (56.0%) patients were male (Table 1). During the one year after the index stroke, 294 patients were diagnosed with depression. The most common morbidities were hypertension (72.4%), dyslipidemia (57.6%), and diabetes (30.0%). About 14% of patients also had a stroke before the index date. About one-third (34.2%) of patients were ever-smokers, and 1.8% had a history of drug abuse or dependence. The majority of the patients had commercial insurance/health maintenance organization (HMO) plans (50.9%) or Medicare (43.4%), and 5.7% of the patients were on Medicaid.

Out of the 5882 patients in the study, 3741 had a complete one-year follow-up without a diagnosis of depression. Compared with patients without the diagnosis of depression, patients with depression were mostly female, younger, and had a history of myocardial infarction, drug abuse or dependence, and a prior stroke. A higher proportion of patients with depression were on Medicaid (Table 1).

### 3.2. One-Year Follow-Up Analysis

In the stratified Cox model, women who suffered from an ischemic stroke had a significantly higher risk for depression (HR = 1.47, 95% CI 1.18–1.85, *p* = 0.001) than men. Medicaid patients had a higher risk for depression (HR = 2.16, 95% CI 1.5–3.12, *p* < 0.001), but Medicare patients did not have a significantly higher hazard compared with patients with commercial/HMO/Medicare/other insurance. A prior history of stroke (HR = 1.58, 95% CI 1.18–2.11, *p* = 0.002) and myocardial infarction (HR = 1.47, 95% CI 1.05–2.06, *p* = 0.025) also showed a significant association with depression within one year of follow-up (Table 2).

**Figure 2:**
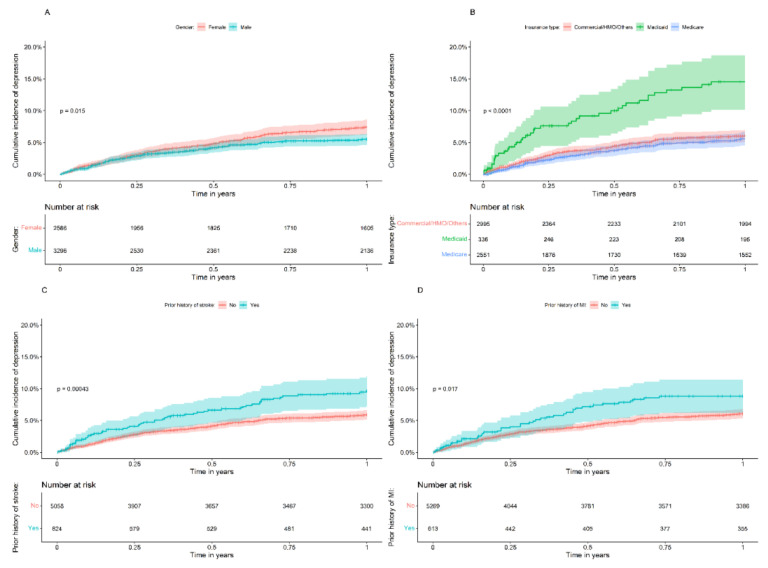
Cumulative incidence of post-stroke depression (PSD) for up to one year stratified by (**A**) gender, (**B**) type of insurance, (**C**) prior history of stroke, and (**D**) history of myocardial infarction.

The cumulative incidence of PSD up to one year was plotted using the Kaplan–Meier estimator for the variables that were significantly associated with PSD in the Cox model. When stratified by gender, the cumulative incidence of PSD was 7.4% (95% CI 6.2–8.5%) in female patients and 5.5% (95% CI 4.7–6.4%) in male patients (Figure 2A). Patients on Medicaid (14.5%, 95% CI 10.2–18.7%) had the highest cumulative incidence of PSD compared with individuals with Medicare (5.6%, 95% CI 4.6–6.6%) and commercial/HMO/other insurance (6.1%, 95% CI 5.1–6.0%, Figure 2B). In the post hoc pairwise log-rank test, the difference in cumulative incidence was significantly higher in Medicaid patients compared with commercial/HMO/other insurance and Medicare plan patients. There was no significant difference between Medicare and commercial/HMO/other insurance plans.

Patients with a history of prior stroke before the index date had a significantly higher PSD incidence (9.6%, 95% CI 7.2–12.0%) compared with those without a prior history of stroke (5.9%, 95% CI 5.1–6.6%, Figure 2C). Similarly, patients with a history of MI had significantly higher PSD incidence (8.8%, 95% CI 6.2–11.4% versus 6.1%, 95% CI 5.4–6.8%, Figure 2D).

## 4. Discussion

Our results showed that female gender, a history of prior stroke, a history of myocardial infarction, and being on Medicaid were significantly associated with PSD in the studied population of patients that suffered an ischemic stroke. Women had 1.47 times higher risk of developing PSD than men. Patients on Medicaid were twice as likely to get PSD than patients on other types of health insurance. Similarly, a history of prior stroke and myocardial infarction increased the risk of PSD by 1.58 and 1.47 times, respectively. Previous studies showed vastly different incidence rates for PSD at different time points. These estimates for PSD include 21% within 12 months [18], 29–31% within 5 years [15,22], 36.5% within 6 weeks [20], and 46% within days [28]. The estimate of 6.4% PSD at one year in our study was lower than other studies. However, comparing results between studies is challenging due to differences in the study design, population, and method of diagnosis of PSD (ICD codes/DSM-5/screening tools or questionnaires).

Based on the Cox model analysis, the female gender was associated with an increased hazard ratio for PSD in our study. Several other studies also showed that women were diagnosed with PSD at higher rates [16,21,24,28,29,30,31,32,33]. The latter could have been due to women having a lower post-stroke health-related quality of life (HRQoL) than men [19]. A lower HRQoL result from women could be attributed to them being less likely to have a caregiver in the time following a stroke. No statistical difference was observed between genders in a study of stroke survivors with caregivers [34]. Although estrogen seems to confer neuroprotection that is lost after menopause, the role of estrogen in PSD is still not established [35]. Estrogen administration was shown to attenuate PSD in animal studies but studies in humans are lacking [36]. It was proposed that estrogen and progesterone might contribute to the higher incidence of depression in women than in men [37]. Thus, the role of estrogen loss in PSD is still debatable. The pathophysiology of PSD is complex since it involves decreased levels of monoamines, abnormal neurotrophic response, dysregulation of the hypothalamic–pituitary–adrenal axis, and glutamate-mediated excitotoxicity [38]. Other hormones, such as thyroid-stimulating hormone, were also implicated in PSD [39].

Younger age at the index stroke date was associated with a higher incidence of PSD or anxiety [9,23,29,32,40], perhaps due to young patients being exposed to higher subjective environmental stressors [40,41]. Other studies found age to be a non-significant risk factor [33,42]. Although in our study, the patients with PSD were significantly younger than patients without PSD within one year of complete follow-up (67.2 versus 71 years, *p* = 0.001), age no longer showed a significant association with PSD in the multivariate Cox model, suggesting it was not an independent predictor. Adding age could not explain the variation in the outcome variable better in the model, suggesting it became stochastic and was withdrawn from the model in the backward elimination process. This age-related discrepancy could be due to the interaction between aging-related biology and psychosocial factors [41].

The association between myocardial infarction and PSD is scarcely mentioned in the literature. Several studies examined depression in post-myocardial infarction. One study found similar rates of depression between patients diagnosed with myocardial infarction and those with stroke [43]. PSD, like other neurodegenerative diseases, may involve disruption of the neurovascular unit or compromised vascular function in the nervous system. It is evident that the endothelium and mural support cell functions go beyond nutrient supply and are instructive and structurally required to allow the entire nervous system to function [44]. Generalized damage to these neurovascular units may affect regular mood processes given that vascular risk factors, such as hypertension, high cholesterol, and diabetes mellitus, are common to both conditions and all are related to depression [43].

Other studies reported dependency, physical disability, and inability to participate in daily life post-stroke as being strongly correlated to PSD [10,11,18,23,24,32,45,46,47]. Stroke severity [10,18,23,42] and cognitive impairment [9,11,16,19,20,47] showed a strong positive association with PSD. Studies also showed positive associations between depression/anxiety and drug abuse/dependence or smoking, with evidence of positive associations in both directions (e.g., smoking exposure associated with later depression and depression at baseline associated with later smoking behavior) [48,49,50,51]. In our study, patients with PSD had higher rates of smoking and drug abuse/dependence but they were not independent predictors in the multivariate Cox model (ever-smoker: HR = 1.21, 95% CI 0.95–1.53, *p* = 0.118; drug abuse/dependence: HR = 1.66, 95% CI 0.87–3.16, *p* = 0.122).

The impact of health insurance on PSD is rarely reported. An epidemiological cohort study in Northwest China showed that a lack of basic health insurance increases the risk of depression [52]. Medicaid expansion in the United States improved access to care and medication, with a significant reduction in the proportion of patients with depression due to lack of health insurance [53,54]. However, compared with patients on commercial, HMO, and other health insurance plans, our study showed that ischemic stroke patients on Medicaid were at higher risk of PSD. Financial stress has a profound impact on human beings in many aspects, including mental and physical health, resulting in unhealthy coping mechanisms. These unhealthy coping mechanisms, in turn, make one’s mental and physical health worse. Low household income increased the risk for several mental disorders and suicide attempts [55]. Depression and anxiety are up to three times as likely for those on low incomes [55].

We examined the relationship between PSD and a variety of potential risk factors. Our results highlighted the need for interdisciplinary care and a strategic approach to stroke patients with known risk factors for depression. Further research is warranted to study the effectiveness of preventative care on developing PSD and recognizing patients who are at risk for mental-health-related illnesses, both before and after a stroke. Research on the association of PSD on quality of life and neuromotor rehabilitation is also warranted to understand the intricate relationships between these entities.

The strength of this study lay in the large sample size with longitudinal data. It is one of the few studies to examine PSD in a rural population. This study also highlighted the association between the type of health insurance and PSD. There were some limitations to this study. First, the retrospective nature of this study did not account for possible confounding variables that were not measured, the absence of measured variables from charts, and potential selection bias due to the nature of the data source. Thus, this study may not generalize to non-rural demographic areas. The use of ICD-9/10-CM codes did not account for patients showing depressive symptoms but not seeking a psychiatric assessment. This approach may lead to an underestimation of the frequency of depression in stroke patients and may explain the lower rate of PSD in this study. Socioeconomic factors [11,23,56], lack of familial or social support [19,20], and lower education level [11,56] were also associated with PSD but could not be considered in this study. The National Institutes of Health Stroke Scale (NIHSS), which was used to assess stroke severity, had a high rate of missingness, and disability data, such as the modified Rankin Scale, was not available, and thus, these two variables could not be included in the analysis.

## 5. Conclusions

Our findings showed that women, patients with a history of prior stroke or myocardial infarction, and patients with Medicaid insurance were more likely to develop PSD. Using an observational study on the EHR data, we confirmed that chronic stress, including financial and health-related stress, irrespective of age, significantly increased the risk for PSD [40]. This study further highlighted the need to screen stroke patients for post-stroke depression using tools such as the PHQ-9. Finally, our findings highlighted the need for the development of a more refined and holistic approach to the care of patients who previously had a stroke or those that may be at higher risk.

## Figures and Tables

**Table 1:** Demographics and clinical profiles of the ischemic stroke patients included in the study.

Variable	Overall	No Depression at One-Year Follow-Up	Depression within One Year Post-Stroke	*p*-Value
Number of patients, *n*	5882	3741	294	
Gender: male, *n* (%)	3296 (56.0)	2136 (57.1)	145 (49.3)	0.011
Age at ischemic stroke diagnosis in years, median [IQR]	72.00 [61.70, 81.20]	71.00 [61.10, 80.10]	67.20 [57.60, 77.88]	0.001
Atrial fibrillation, *n* (%)	1274 (21.7)	759 (20.3)	51 (17.3)	0.256
Hypertension, *n* (%)	4261 (72.4)	2763 (73.9)	215 (73.1)	0.838
Myocardial infarction, *n* (%)	613 (10.4)	355 (9.5)	41 (13.9)	0.018
Diabetes, *n* (%)	1762 (30.0)	1134 (30.3)	90 (30.6)	0.967
Dyslipidemia, *n* (%)	3387 (57.6)	2288 (61.2)	175 (59.5)	0.623
Congestive heart failure, *n* (%)	708 (12.0)	364 (9.7)	35 (11.9)	0.271
Hypercoagulable states, *n* (%)	76 (1.3)	49 (1.3)	5 (1.7)	0.766
Chronic liver disease, *n* (%)	107 (1.8)	67 (1.8)	4 (1.4)	0.756
Chronic lung disease, *n* (%)	1028 (17.5)	657 (17.6)	49 (16.7)	0.757
Rheumatic diseases, *n* (%)	201 (3.4)	125 (3.3)	14 (4.8)	0.263
Chronic kidney disease, *n* (%)	928 (15.8)	554 (14.8)	40 (13.6)	0.635
Neoplasm, *n* (%)	850 (14.5)	532 (14.2)	43 (14.6)	0.917
Perivascular disease, *n* (%)	834 (14.2)	548 (14.6)	44 (15.0)	0.950
Ever-smoker, *n* (%)	2013 (34.2)	1397 (37.3)	123 (41.8)	0.142
Drug abuse or dependence, *n* (%)	103 (1.8)	51 (1.4)	10 (3.4)	0.012
Insurance, *n* (%)				<0.001
Commercial/HMO/others	2995 (50.9)	1994 (53.3)	149 (50.7)	
Medicaid	336 (5.7)	195 (5.2)	39 (13.3)	
Medicare	2551 (43.4)	1552 (41.5)	106 (36.1)	
Stroke history, *n* (%)	824 (14.0)	441 (11.8)	58 (19.7)	<0.001

**Table 2:** Cox proportional hazards model for post-stroke depression (PSD).

Variable	Hazard Ratio (HR)	95% Confidence Interval (CI)	*p*-Value
Gender: female	1.47	1.18–1.85	0.001
Insurance type:			
commercial/HMO/others	Reference		
Medicaid	2.16	1.5–3.12	<0.001
Medicare	0.95	0.73–1.23	0.692
History of prior stroke	1.58	1.18–2.11	0.002
History of drug abuse/dependence	1.66	0.87–3.16	0.122
Prior history of myocardial infarction	1.47	1.05–2.06	0.025
Ever-smoker	1.21	0.95–1.53	0.118

## Data Availability

The data used in the study is not publicly available. The data may be made available via a data-sharing agreement with Geisinger. The corresponding author may be contacted for a data-sharing request.
